# Constitutive expression of selected genes from the pentose phosphate and aromatic pathways increases the shikimic acid yield in high-glucose batch cultures of an *Escherichia coli* strain lacking PTS and *pykF*

**DOI:** 10.1186/1475-2859-12-86

**Published:** 2013-09-30

**Authors:** Alberto Rodriguez, Juan A Martínez, José L Báez-Viveros, Noemí Flores, Georgina Hernández-Chávez, Octavio T Ramírez, Guillermo Gosset, Francisco Bolivar

**Affiliations:** 1 Departamento de Ingeniería Celular y Biocatálisis, Instituto de Biotecnología, Universidad Nacional Autónoma de México (UNAM), Apdo. Postal 510-3, Cuernavaca, Morelos 62250, Mexico; 2 Centro de Investigación en Biotecnología, Universidad Autónoma del Estado de Morelos (UAEM), Av. Universidad 2000, Cuernavaca, Morelos 62250, Mexico; 3 Departamento de Medicina Molecular y Bioprocesos, Instituto de Biotecnología, Universidad Nacional Autónoma de México (UNAM), Apdo. Postal 510-3, Cuernavaca, Morelos 62250, Mexico

**Keywords:** Shikimic acid, Synthetic operon, *Escherichia coli*, *pykF*, Aromatic compounds

## Abstract

**Background:**

During the last two decades many efforts have been directed towards obtaining efficient microbial processes for the production of shikimic acid (SA); however, feeding high amounts of substrate to increase the titer of this compound has invariably rendered low conversion yields, leaving room for improvement of the producing strains. In this work we report an alternative platform to overproduce SA in a laboratory-evolved *Escherichia coli* strain, based on plasmid-driven constitutive expression of six genes selected from the pentose phosphate and aromatic amino acid pathways, artificially arranged as an operon. Production strains also carried inactivated genes coding for phosphotransferase system components (*ptsHIcrr*), shikimate kinases I and II (*aroK* and *aroL*), pyruvate kinase I (*pykF*) and the lactose operon repressor (*lacI*).

**Results:**

The strong and constitutive expression of the constructed operon permitted SA production from the beginning of the cultures, as evidenced in 1 L batch-mode fermentors starting with high concentrations of glucose and yeast extract. Inactivation of the *pykF* gene improved SA production under the evaluated conditions by increasing the titer, yield and productivity of this metabolite compared to the isogenic *pykF*^+^ strain. The best producing strain accumulated up to 43 g/L of SA in 30 h and relatively low concentrations of acetate and aromatic byproducts were detected, with SA accounting for 80% of the produced aromatic compounds. These results were consistent with high expression levels of the glycolytic pathway and synthetic operon genes from the beginning of fermentations, as revealed by transcriptomic analysis. Despite the consumption of 100 g/L of glucose, the yields on glucose of SA and of total aromatic compounds were about 50% and 60% of the theoretical maximum, respectively. The obtained yields and specific production and consumption rates proved to be constant with three different substrate concentrations.

**Conclusions:**

The developed production system allowed continuous SA accumulation until glucose exhaustion and eliminated the requirement for culture inducers. The obtained SA titers and yields represent the highest reported values for a high-substrate batch process, postulating the strategy described in this report as an interesting alternative to the traditionally employed fed-batch processes for SA production.

## Background

**Figure 1:**
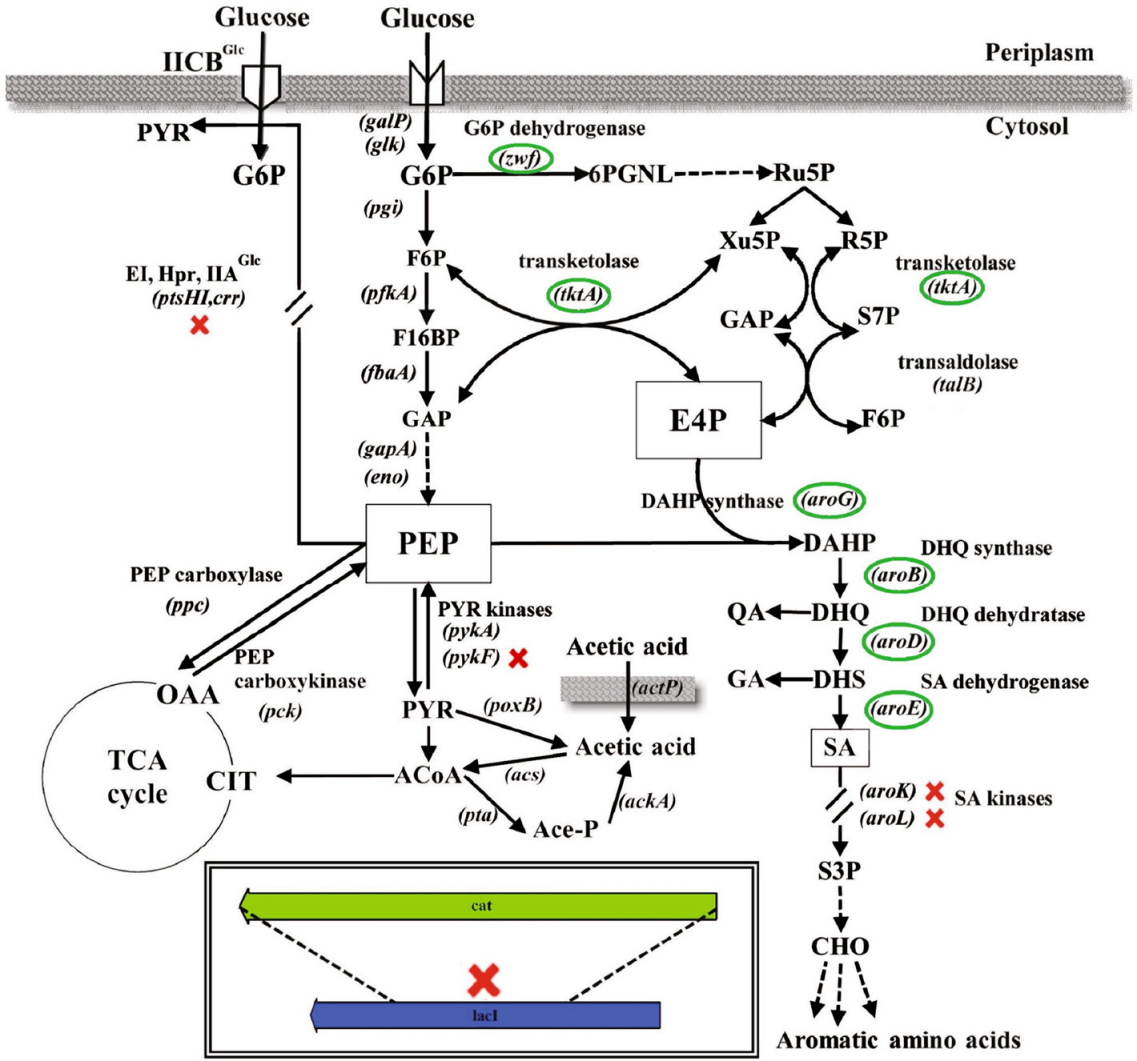
**Genetic modifications applied in this report to enhance the production of shikimic acid from glucose in the laboratory-evolved *E. coli *strain PB12.** Inactivated genes are indicated with a cross and plasmid-expressed genes are circled (see Methods for details). Dashed arrows indicate more than one catalytic step. G6P = glucose 6-phosphate; F6P = fructose 6-phosphate; GAP = glyceraldehyde 3-phosphate; 6PGNL = 6-phosphogluconolactone; Ru5P = ribulose 5-phosphate; R5P = ribose 5-phosphate; Xu5P = xylulose 5-phosphate; S7P = sedoheptulose 7-phosphate; E4P = erythrose 4-phosphate; PEP = phosphoenolpyruvate; PYR = pyruvate; ACoA = acetyl-coenzyme A; Ace-P = acetyl phosphate; CIT = citrate; OAA = oxaloacetate; DAHP = 3-deoxy-D-arabinoheptulosonate 7-phosphate; DHQ = 3-dehydroquinic acid; DHS = 3-dehydroshikimic acid; QA = quinic acid; GA = gallic acid; SA = shikimic acid; S3P = shikimate 3-phosphate; CHO = chorismate; IICBGlc = membrane component of glucose-specific PTS permease; E1 = PTS enzyme 1; Hpr = PTS histidine protein; IIAGlc = cytosolic component of glucose-specific PTS permease. Genes coding for enzymes not named in the figure: *galP*, galactose permease; *glk*, glucokinase; *pgi*, phosphoglucose isomerase; *pfkA*, 6-phosphofructokinase I; *fbaA*, fructose bisphosphate aldolase class II; *gapA*, glyceraldehyde 3-phosphate dehydrogenase; *eno*, enolase; *actP*, acetate permease; *acs*, acetyl-coenzyme A synthetase; *pta*, phosphate acetyltransferase; *ackA*, acetate kinase; *poxB*, pyruvate oxidase.

Shikimic acid (SA) is an intermediate compound in the aromatic amino acid (AAA) biosynthetic pathway in plants and bacteria (Figure 1). This metabolite is utilized as starting material in the chemical synthesis of oseltamivir phosphate (Tamiflu), used for influenza treatment [1-3]. Several genetic strategies have been reported for improving SA productivity and yield in *Escherichia coli*. These strategies aim to increase the availability of the direct precursors of the AAA pathway, erythrose 4-phosphate (E4P) and phosphoenolpyruvate (PEP), by genetic alterations that promote a convenient redistribution of carbon fluxes in the central metabolism [4,5]. Complementary approaches include the interruption of the AAA pathway after SA formation by inactivation of the genes coding for shikimate kinases (*aroK* and *aroL*), as well as enhancements in carbon channeling towards SA by overexpression of feedback-resistant DAHP synthases, shikimate dehydrogenase, transketolase, and DHQ synthase enzymes (coded by *aroFGH*^fbr^, *aroE*, *tktA*, and *aroB*, respectively) (Figure 1) [6-9]. In an attempt to further increase the intracellular availability of PEP, strains overexpressing PEP synthase (coded by *ppsA*), or lacking the PEP:carbohydrate phosphotransferase system (PTS) and the pyruvate kinase isozymes (coded by *pykF* and *pykA*), have also been evaluated (Figure 1) [10-13].

Although the implementation of these modifications along with bioengineering strategies has led to diverse *E. coli* strains capable of accumulating SA, the yields obtained to date are still far from the theoretical maximum [10,11,14,15]. This can be partially attributed to the fact that most expression systems used involve genes controlled by a mixture of inducible and native promoters of variable strengths, contained in more than one type of plasmid. These imbalances often cause a metabolic burden and heterogeneities on the intensity and temporality of gene expression, which may translate into suboptimal production capabilities of the recombinant strains, resulting in low productivity and yield of SA [16-19]. Consequently, optimized DNA expression systems and genetic backgrounds are needed for promoting a more efficient carbon channeling towards SA formation.

With the goal of producing aromatic compounds, our group has constructed and characterized strains lacking PTS, the major glucose transport system [20]. One of such strains is PB11, which grows poorly on glucose due to the inactivation of PTS [21,22]. Strain PB12, a derivative of PB11 with a 400% increased growth rate, was isolated in a short laboratory adaptive evolution process to foster derivatives growing in glucose [21,22]. This strain can simultaneously utilize glucose and other carbon sources (acetate, glycerol and various carbohydrates) in minimal medium due to the lack of catabolite repression exerted by PTS [21,23]. Whole genome analysis allowed the identification of the genetic changes that occurred in PB12, suggesting that the deletion of 12 genes, including *rppH*, *galR* and *mutH*, is the main reason for its rapid growth on glucose [24].

It was reported that PB12, which assimilates glucose by the non-PTS symporter GalP [25], can be engineered to accumulate SA in culture media containing glucose (Glc) and yeast extract (YE). For instance, when PB12 was transformed with two plasmids encoding four biosynthetic genes, the variant with both functional pyruvate kinases accumulated the highest SA concentration (up to 7 g/L), but the highest yield of aromatic compounds was achieved by a derivative with an inactivated *pykF* gene [11]. This result may be related to other reported effects caused by the inactivation of *pykF*, such as an increase in plasmid copy number per cell [26], low acetate production due to less glycolytic overflux [13,27,28], or higher concentrations of the AAA pathway enzymes [29]. Interestingly, in spite of the aforesaid features that can be beneficial for SA production, the metabolic engineering efforts to overproduce this compound have been mainly applied to strains with a *pykF*^+^ background, probably because of their typically higher glucose consumption rates compared to the *pykF*^-^ counterparts [11,30].

**Figure 2:**
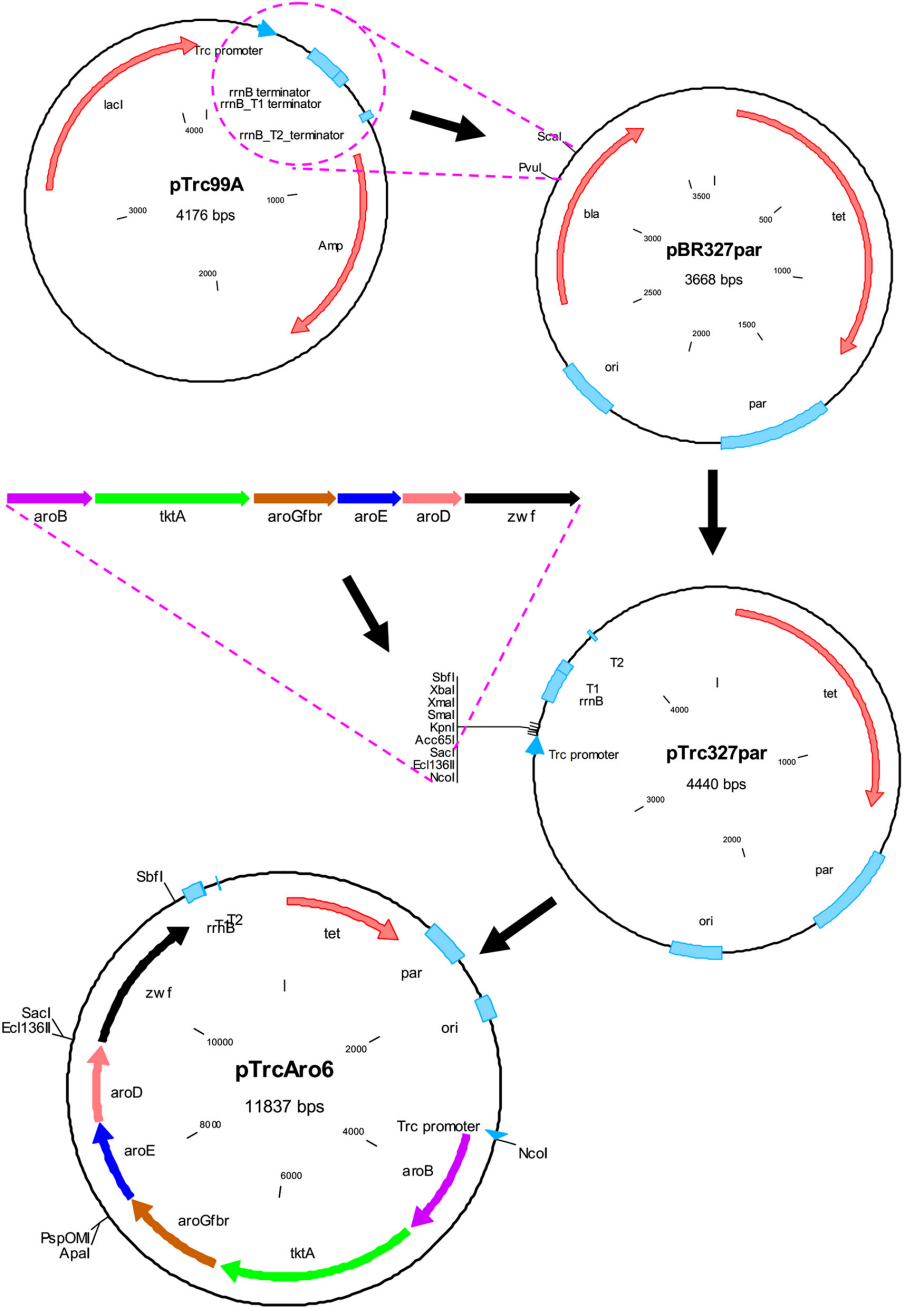
**Simplified scheme of the steps required in the construction of plasmid pTrcAro6, carrying 6 synthetic genes under the control of the Trc promoter.** First, a segment of pTrc99A was amplified and ligated into pBR327par, creating plasmid pTrc327par. A synthetic operon comprising the required genes (*aroB, tktA, aroG*^*fbr*^, *aroE, aroD*, and *zwf*) was assembled separately and transferred to pTrc327par, generating pTrcAro6. The dotted lines indicate the site and orientation of some of the performed ligation reactions. Only the relevant restriction sites are displayed. A more detailed scheme of the constructions is presented in Additional file 3.

Here, we propose that a PTS^-^*pykF*^-^ strain has the potential to increase the yield and titer of SA when compared to an isogenic *pykF*^*+*^ strain, provided that the gene expression system permits an appropriate temporal coordination in the synthesis of the enzymes required to channel the carbon towards SA, while reducing the accumulation of acetate and intermediate compounds in the AAA pathway. In order to accomplish this goal a synthetic operon was constructed containing the coding sequences of six genes selected from the pentose phosphate (PPP) and AAA pathways (Figure 1), controlled by a single constitutive Trc promoter [31] and inserted it into a high-copy plasmid containing a region that confers segregational stability (Figure 2) [32]. The resulting plasmid was transformed into a modified PB12 strain with inactive *aroK*, *aroL*, *pykF*, and *lacI* genes, and was cultured in fermentors using mineral media containing Glc and supplemented with YE. Overall, the strategy proposed in this report allowed the overproduction of SA from the beginning of the culture, resulting in a high titer and yield of SA with relatively low accumulation of acetate and aromatic byproducts. It was also found that, under the high-substrate conditions tested, the SA titer was independent of the YE concentration and the maximum biomass produced depended exclusively on the initial YE concentration but not on the amount of glucose.

## Results and discussion

### Construction of strains derived from PB12 *aroK*^-^*aroL*^-^ containing a plasmid designed for the constitutive expression of a synthetic operon used in the production of shikimic acid

Unpublished evidence from our laboratory indicates that the production of aromatic compounds in the laboratory-evolved strain PB12 can attain higher levels when the transcriptional induction of the genes involved in canalizing carbon flux into the AAA pathway occurs at the beginning of fermentations. Taking into account this observation, a new strategy was developed for optimizing the production of SA in PB12 carrying inactive *aroK* and *aroL* genes (Figure 1). This strategy included the design and construction of a plasmid for the strong and stable expression of six key genes arranged in the form of a synthetic operon, controlled exclusively by a single Trc promoter. In order to reduce metabolic burden, a single plasmid derived from pBR327 carrying the *par* locus for increased plasmid stability was utilized as the vector [32], after incorporating a fragment containing the promoter, polylinker, and transcriptional terminators from pTrc99A (Figure 2).

The initial part of the operon was constructed by sequential amplification and ligation of the first 4 coding sequences (*aroB, tktA, aroG*^fbr^, and *aroE*) into the polylinker of plasmid pBRINT-Ts Cm, used as a cloning scaffold (see Methods). Later, the 4-gene construction was transferred to the hybrid plasmid pTrc327par in conjunction with 2 more genes (*aroD* and *zwf*), leading to an 8Kb operon contained in a 12Kb plasmid (Figure 2). The resulting plasmid, termed pTrcAro6, was transformed into the PB12 *aroK*^-^*aroL*^-^ strain devoid of the *lacI*^q^ gene, allowing constitutive expression of the genes of interest (Table 1). For simplicity, the generated PB12 *aroK*^-^*aroL*^-^*lacI*^-^ strain was termed AR2. After the *pykF* gene was inactivated in AR2, the resulting strain was named AR3. Strains derived from AR2 and AR3 carrying plasmid pTrcAro6 were named AR26 and AR36, respectively (Table 1).

**Table 1:** ***Escherichia coli *strains and plasmids utilized in this report**

**Strains**		
**Name**	**Characteristics**	**Reference**
JM101	F´ *tra*D36 *proA* ^+^ *proB* ^+^ *lacI*q *lac*ZΔM15/*supE thi* Δ(*lac*-*proAB*) *rpoS(33 am)*	Messing [41]
PB11	JM101 Δ(*ptsH, ptsI, crr)*::*kan*	Flores et al. [21]; Flores et al. [22]
PB12	PB11, PTS^-^ Glc^+^; laboratory-evolved strain	Flores et al. [21]; Flores et al. [22]
AR2	PB12 *lacI*^-^*aroK*^-^*aroL*^-^	This work
AR3	PB12 *lacI*^-^*aroK*^-^*aroL*^-^*pykF*^-^	This work
AR26	AR2 + pTrcAro6 (Trc/*aroB*^*+*^*tktA*^*+*^*aroGfbr*^*+*^*aroE*^*+*^*aroD*^*+*^*zwf*^*+*^)	This work
AR36	AR3 + pTrcAro6 (Trc/*aroB*^*+*^*tktA*^*+*^*aroGfbr*^*+*^*aroE*^*+*^*aroD*^*+*^*zwf*^*+*^)	This work
AR2e	AR2 + pTrc327par (plasmid vector without synthetic operon)	This work
AR3e	AR3 + pTrc327par (plasmid vector without synthetic operon)	This work
**Plasmids**		
**Name**	**Characteristics**	**Reference**
pKD3	PCR template for amplification of chloramphenicol resistance gene flanked by homologous sequences	Datsenko and Wanner [42]
pKD46	Plasmid expressing λ-Red recombinase system with thermosensitive origin of replication	Datsenko and Wanner [42]
pCP20	FLP recombinase expression plasmid	Cherepanov and Wackernagel [43]
pBR327par	Derivative of pBR322 exhibiting increased copy number and segregational stability	Zurita et al. [32]
pTrc99A	Multipurpose expression plasmid bearing *lacI* gene and a polylinker in front of Trc promoter	Amann et al. [31]
pTrc327par	Contains the promoter, polylinker, and terminators of pTrc99A, and *par* and *ori* regions of pBR327par	This work (Figure 2)
pTrcAro6	pTrc327par containing a 6-gene synthetic operon to enhance the production of shikimate	This work (Figure 2)

The spatial arrangement of the coding sequences that constitute the synthetic operon in pTrcAro6, flanked by the Trc promoter and transcriptional terminators, is shown in Figure 2. *aroB* is the first gene in the operon since several evidences indicate that its low expression is one of the limiting steps in the production of aromatic compounds [33-35]. Plasmid pTrcAro6 also carries the *tktA* and *aroG*^fbr^ genes, whose products are involved in E4P synthesis and its condensation with PEP to form DAHP, the first aromatic compound (Figure 1). *aroD* and *aroE* genes were also included to promote an efficient conversion of DHQ to SA. Additionally, this plasmid carries the *zwf* gene, coding for the first enzyme of the PPP (Figure 1). The decision to include this gene was based on the following observations: 1) the overexpression of *zwf* substantially recovered the growth rate loss due to plasmid metabolic load in strain JM101 growing on glucose as only carbon source [36]; 2) it has been reported that strain PB12 displays a particularly low carbon flux partition at the glucose 6-phosphate (G6P) node towards the PPP (5% of the consumed G6P compared to 22% in the parental strain JM101) [25]. Therefore, an overexpression of this gene should increase NADPH availability, required in catalytic amounts by the enzyme shikimate dehydrogenase (AroE), and may alleviate potential growth affectations by redirecting more G6P towards nucleotide and amino acid biosynthesis in strains derived from PB12 [37]. However, the experiments presented in this report did not aim to dissect the specific effect of any utilized gene but instead sought to characterize the consequences of expressing all of them as an operon.

In order to promote an efficient translation of every gene, each coding sequence was amplified using designated primers that introduced a consensus Shine-Dalgarno sequence located 8 bp upstream of the translation start site. The nucleotide sequence of the constructed operon is presented in Additional file 1.

### Assessment of the effects caused by *pykF* inactivation in strains expressing the Aro6 operon

To evaluate the effects caused by *pykF* inactivation on the production of SA, the performance of production strains AR26 (*pykF*^+^) and AR36 (*pykF*^-^) was compared using shake flasks containing 15 g/L of Glc and 5 g/L of YE. As a control, the same strains containing an empty pTrc327par plasmid (without the Aro6 operon), AR2e and AR3e, were also included.

**Figure 3:**
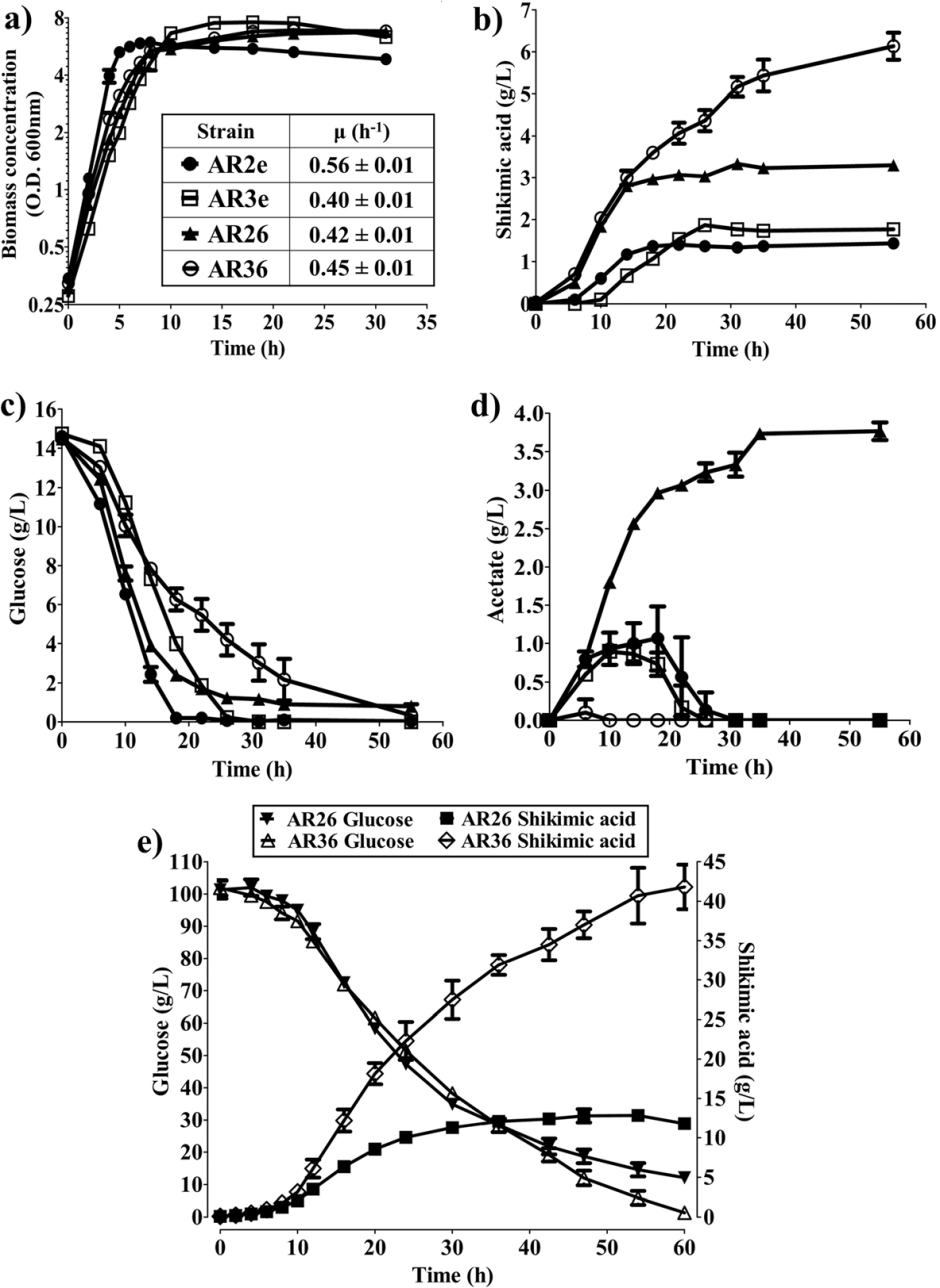
**Behavior of strains AR26, AR36, and their empty-plasmid derivatives, AR2e (*pykF***^**+**^**) and AR3e (*pykF***^***-***^**), using shake flasks containing 15 g/L of Glc and 5 g/L of YE (a,b,c,d), and 1 L fermentors containing 100 g/L of Glc and 15 g/L of YE (e). a)** Growth; **b)** SA production; **c)** Glc consumption; **d)** acetate production; **e)** Glc consumption and SA production of AR26 and AR36 in fermentors. Error bars represent standard deviation.

Even though SA accumulated in all cases, as expected for mutants in *aroK* and *aroL*, the strains containing pTrcAro6 reached higher SA concentrations than the ones with an empty plasmid (Figure 3b). Moreover, the SA titer was almost two times higher in AR36 than in AR26 (6.1 g/L vs. 3.3 g/L). A decrease in Glc consumption was observed in strain AR26 after approximately 18 h of culture, correlating with high acetate concentration and an arrest in the production of SA. In contrast, strain AR36 exhibited constant Glc consumption and negligible amounts of acetate were produced (Figure 3c, 3d). These results demonstrate that the genes present in the artificial operon are functional and promote the production of SA since the beginning of the culture. Their constitutive expression diminished the specific growth rate (μ) by 25% in the *pykF*^+^ background, and marginally increased it in the *pykF*^-^ variant, but did not cause significant changes to the maximum biomass produced (X_max_) compared to strains with an empty plasmid (Figure 3a). Remarkably, in the operon-expressing strains under these growth conditions, the inactivation of the *pykF* gene increased the production of SA, eliminated the accumulation of acetate, and allowed steady Glc consumption.

To determine if the higher acetate production and lower SA production in AR26 compared to AR36 is a consequence of the inherently low oxygen availability and acidification of the medium in shake flask cultures, both strains were cultured in 1 L batch fermentors under controlled conditions of pH and dissolved oxygen tension (DOT). As an approach to increase the SA titer, the initial concentration of Glc in these experiments was raised to 100 g/L, and the YE concentration was concomitantly increased to 15 g/L to allow higher biomass generation.

Under these conditions strain AR36 produced 42 g/L of SA in 60 h, consuming all the Glc, and accumulating 12 g/L of acetate. In contrast, after 47 h strain AR26 produced a maximum of 13 g/L of SA, did not exhaust the Glc, and accumulated 29 g/L of acetate (Figure 3e and Table 2). Regardless of the controlled conditions in the fermentors, where the pH was kept at 7 and the DOT was higher than 20% at all times, the production profiles of both strains resembled the behavior observed in shake flasks, with AR26 producing more acetate and less SA. Even when the global volumetric Glc consumption rate (Qs_global_), μ and X_max_ attained by both strains were similar, the productivity, yield, and titer were more than twofold higher in AR36 than in AR26 (Figure 3e and Table 2).

**Table 2:** Comparative data from 1 L batch fermentations of strains AR26 and AR36, using 100 g/L of Glc and 15 g/L of YE as substrates

		
**Strain**	AR26	AR36
**SA titer (g/L)**	12.95 ± 0.64	41.80 ± 2.83
**Glc consumed (g/L)**	82.65 ± 4.88	103.70 ± 6.79
**Duration of culture (h)**	47	60
**Y**_**SA/Glc **_**(mol/mol)**	0.16 ± 0.02	0.42 ± 0.00
**Acetate titer (g/L)**	29.35 ± 0.21	11.90 ± 0.14
**X**_**max **_**(g/L)**	6.18 ± 0.10	6.54 ± 0.09
**μ (h**^**-1**^**)**	0.45 ± 0.01	0.45 ± 0.02
**Qp**_**global **_(gSA/L*h)	0.27 ± 0.02	0.75 ± 0.07
**Qs**_**global **_(gGlc/L*h)	-1.76 ± 0.10	-1.73 ± 0.11

Y_SA/Glc_ = SA yield from Glc; X_max_ = maximum attained biomass expressed as dry cell weight; Qp_global_ = volumetric SA productivity for the entire fermentation; Qs_global_ = volumetric Glc consumption rate for the entire fermentation; μ = specific growth rate.

It is remarkable that such large differences in acetate and SA production were observed by disrupting only one gene, which demonstrates the advantages of the combined inactivation of PTS and *pykF* when using a constitutive expression system in an evolved *E. coli* strain. To account for the observed improvements in SA production, we suggest that the early and constant expression of enzymes encoded in the operon could maintain a steady consumption of glycolytic intermediates throughout the cultures, preventing high fluctuations in their intracellular concentrations. We hypothesize that the combination of this steady metabolic state with a reduced flux from PEP to pyruvate caused by the inactivation of the *pykF* gene may increase the availability of PEP and other glycolytic precursors for SA production without decreasing the Glc consumption rate. However, we acknowledge that in the absence of measured intracellular metabolite concentrations, these remarks are speculative.

### Fermentation profiles of AR36 in batch cultures

Taking into account the previous results, AR36 was selected for further characterization of its kinetic and stoichiometric performance in 1 L fermentors. To accomplish such purpose, the production of SA was tested with three different high-substrate culture conditions. Growth, Glc and byproducts were measured for each case, which in turn allowed a comparison of the productivities and yields.

**Figure 4:**
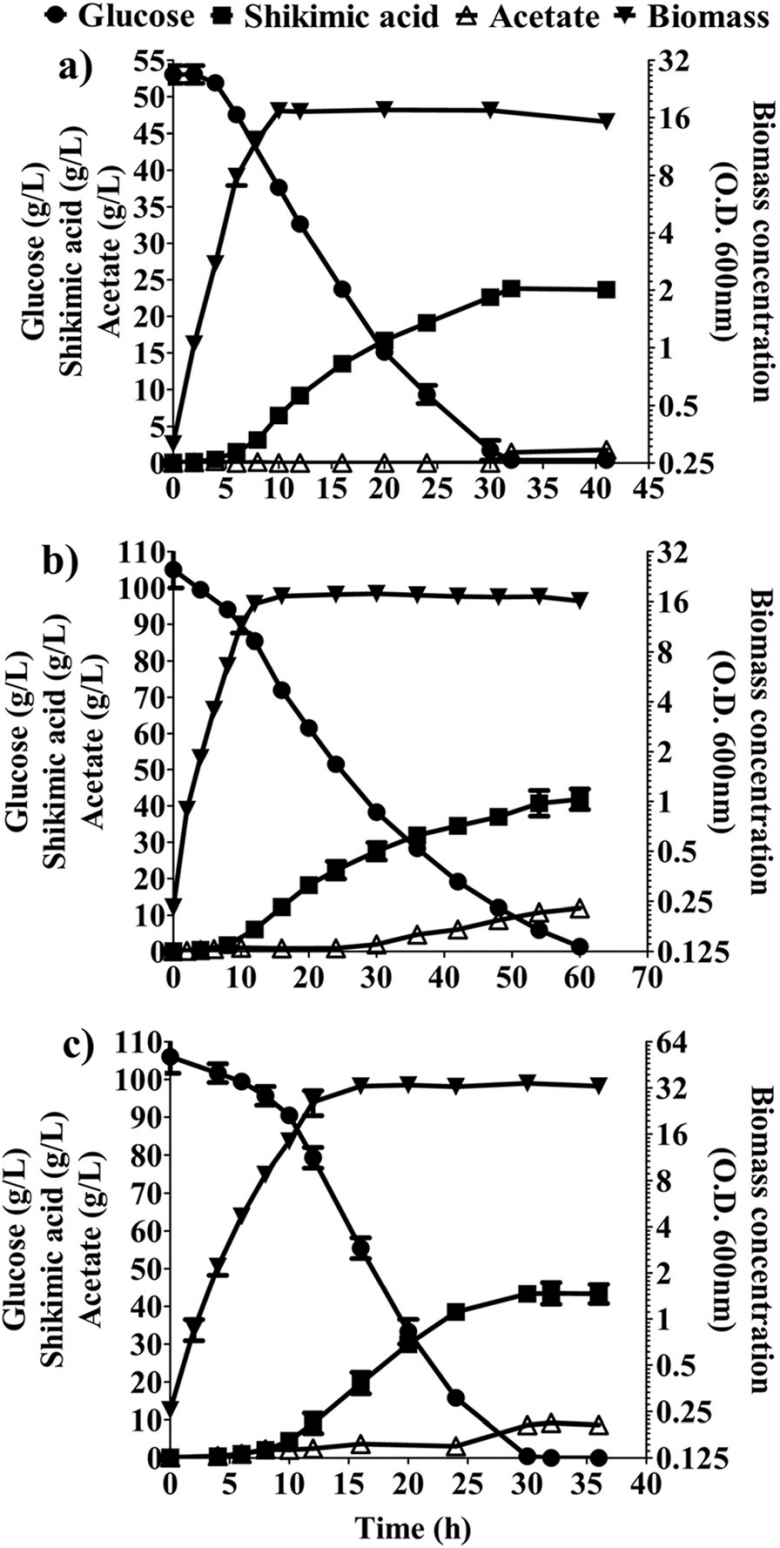
**Fermentation profile of strain AR36 cultivated in 1 L bioreactors with three different substrate concentrations. a)** 50 g/L of Glc and 15 g/L of YE; **b)** 100 g/L of Glc and 15 g/L of YE; **c)** 100 g/L of Glc and 30 g/L of YE. Glc: circles; SA: squares; acetate: open triangles; biomass concentration: inverted triangles. Error bars represent standard deviation.

**Figure 5:**
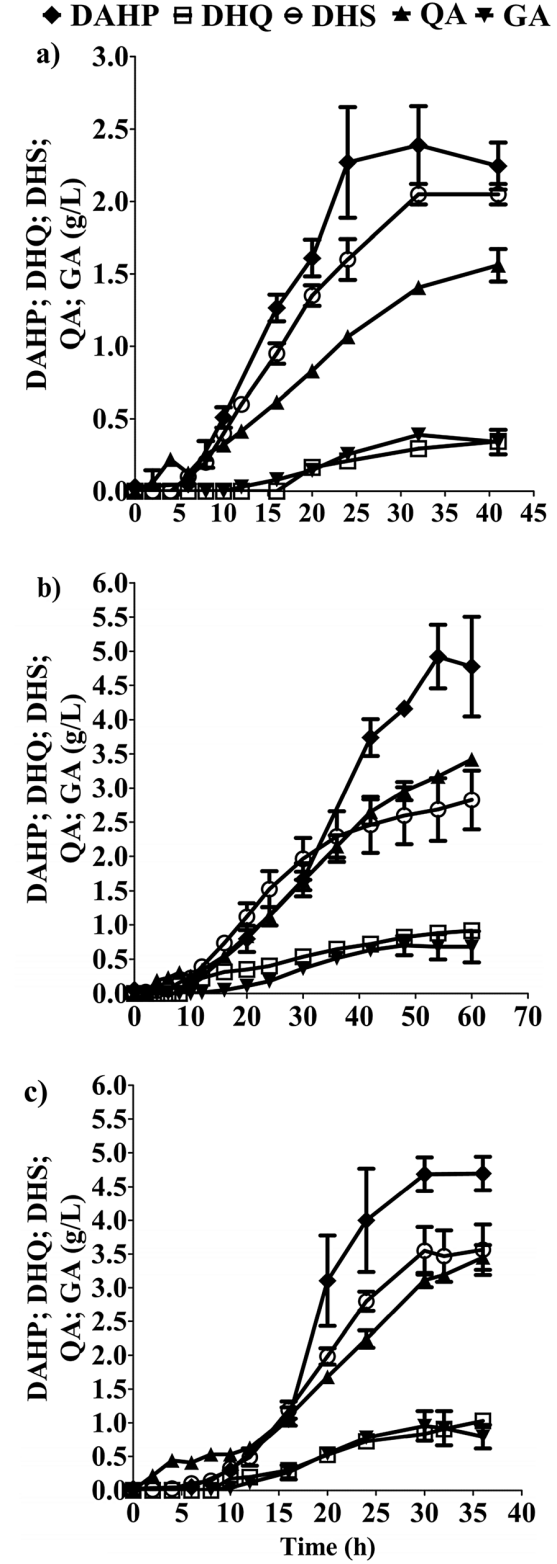
**Aromatic byproducts of the SA pathway detected in 1 L fermentor cultures of strain AR36 using three different substrate concentrations. a)** 50 g/L of Glc and 15 g/L of YE; **b)** 100 g/L of Glc and 15 g/L of YE; **c)** 100 g/L of Glc and 30 g/L of YE. Diamonds: DAHP (3-deoxy-D-arabinoheptulosonate 7-phosphate); squares: DHQ (3-dehydroquinic acid); circles: DHS (3-dehydroshikimic acid); triangles: QA (quinic acid); inverted triangles: GA (gallic acid). Error bars represent standard deviation.

First, 50 g/L of Glc and 15 g/L of YE were utilized (Figure 4a). Growth occurred during the first 10 h, generating 6.3 g/L of dry cell weight with a μ of 0.53 h^-1^. Under this condition, 24 g/L of SA were produced in 32 h. Glc consumption and SA production occurred since the beginning of the fermentation and lasted until Glc exhaustion, although the specific Glc consumption rate (qs) and specific SA productivity (qp) were higher in exponential phase (Table 3). The resulting yield of SA on Glc (Y_SA/Glc_) was 0.47 mol/mol and the global volumetric SA productivity (Qp_global_) was 0.74 gSA/L*h (Table 3). With respect to the accumulation of byproducts in the SA pathway, concentrations of 2.4 g/L of DAHP, 2.1 g/L of DHS, 1.4 g/L of QA, 0.4 g/L of GA, and 0.3 g/L of DHQ, were present in the supernatant at the end of the fermentation (Figure 5a). Under these conditions, virtually no acetate was produced during the course of the fermentation, reaching a maximum concentration of 1.5 g/L after 32 h (Figure 4a).

**Table 3:** Comparison of measured metabolites and calculated kinetic and stoichiometric parameters between three fermentations of strain AR36 with different substrate concentrations

			
**Strain**	AR36	AR36	AR36
**Culture conditions**	Batch 50 g/L Glc + 15 g/L YE	Batch 100 g/L Glc + 15 g/L YE	Batch 100 g/L Glc + 30 g/L YE
**SA titer (g/L)**	23.80 ± 0.00	41.80 ± 2.83	43.30 ± 0.57
**Glc consumed (g/L)**	52.65 ± 1.20	103.70 ± 6.79	105.55 ± 4.45
**Duration of culture (h)**	32	60	30
**Y**_**SA/Glc **_**(mol/mol)**	0.47 ± 0.01	0.42 ± 0.00	0.42 ± 0.01
**Acetate titer (g/L)**	1.45 ± 0.00	11.90 ± 0.14	8.65 ± 0.92
**X**_**max **_**(g/L)**	6.30 ± 0.09	6.54 ± 0.09	12.54 ± 0.06
**μ (h**^**-1**^**)**	0.53 ± 0.03	0.45 ± 0.02	0.45 ± 0.00
**Qp**_**global **_(gSA/L*h)	0.74 ± 0.00	0.75 ± 0.07	1.44 ± 0.02
**Qs**_**global **_(gGlc/L*h)	-1.65 ± 0.04	-1.74 ± 0.11	-3.52 ± 0.15
**qp**_**exp **_(gSA/gDCW*h)	0.38 ± 0.00	0.34 ± 0.03	0.46 ± 0.06
**qs**_**exp **_(gGlc/gDCW*h)	-1.14 ± 0.15	-1.11 ± 0.00	-1.18 ± 0.03
**qp**_**sta **_(gSA/gDCW*h)	0.16 ± 0.01	0.19 ± 0.02	0.20 ± 0.01
**qs**_**sta **_(gGlc/gDCW*h)	-0.36 ± 0.01	-0.42 ± 0.01	-0.42 ± 0.03

Y_SA/Glc_ = SA yield from Glc; X_max_ = maximum attained biomass expressed as dry cell weight; Qp_global_ = volumetric SA productivity for the entire fermentation; Qs_global_ = volumetric Glc consumption rate for the entire fermentation; qp_exp_ = specific SA productivity for exponential phase; qs_exp_ = specific Glc consumption rate for exponential phase; qp_sta_ = specific SA productivity for stationary phase; qs_sta_ = specific Glc consumption rate for stationary phase; μ = specific growth rate.

**Figure 6:**
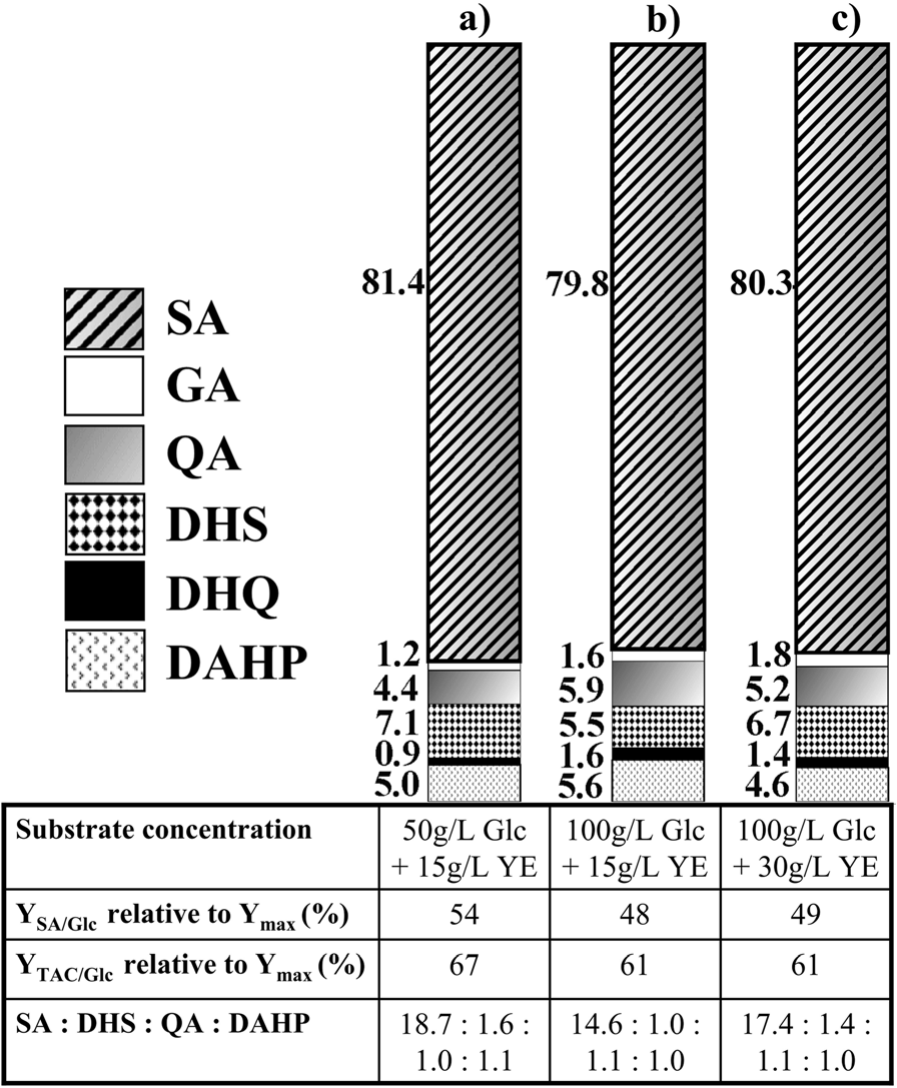
**Molar percentage of each aromatic compound produced in strain AR36 with respect to the total in batch cultures starting with: a) 50 g/L of Glc and 15 g/L of YE; b) 100 g/L of Glc and 15 g/L of YE; c) 100 g/L of Glc and 30 g/L of YE.** Calculated yields and molar ratios of the produced aromatic compounds are shown below each bar. The comparisons were made with the concentrations measured in the supernatant at the end of the fermentations. Y_SA/Glc_ = yield of SA from Glc; Y_TAC/Glc_ = yield of total aromatic compounds from Glc; Y_max_ = maximum theoretical yield of aromatic compounds.

Considering that 50 g/L of Glc were consumed completely, a second batch experiment was initiated with 100 g/L of Glc and 15 g/L of YE. As stated in the comparison with AR26 in the previous section, AR36 grown under these conditions produced approximately 42 g/L of SA in 60 h (Figure 4b). In this case, after consuming about 100 g/L of glucose and attaining the maximum concentration of SA, the strain produced 12 g/L of acetate. The values obtained for Y_SA/Glc_, Qp_global_, Qs_global,_ X_max_, and μ, were similar to those obtained with 50 g/L of Glc and 15 g/L of YE (Table 3). These experiments show that when using the same YE concentration, twice the amount of Glc is consumed in almost twice the time, indicating that the average glucose consumption rate is maintained between both culture conditions. Concentrations of 4.8 g/L of DAHP, 2.8 g/L of DHS, 3.4 g/L of QA, 0.7 g/L of GA, and 0.9 g/L of DHQ, were present in the supernatant after 60 h (Figure 5b). Interestingly, when doubling the Glc concentration the intermediate products of the AAA pathway increased in a fairly proportional manner with the SA, indicating that the consumption of 100 g/L of Glc did not apparently generate new carbon flux bottlenecks. As a result, the amount of SA formed with respect to the total aromatic compounds produced was close to 80% in both experiments (Figure 6).

The effect of increasing the YE on SA productivity was investigated with a third set of experiments, using 100 g/L of Glc and 30 g/L of YE. Although the biomass was doubled when using twice the concentration of YE, the SA titer, μ and Y_SA/Glc_ were very similar to those obtained in the culture with 100 g/L of Glc and 15 g/L of YE (Figure 4b and Figure 4c). In conjunction with data obtained from the other two conditions, these findings suggest that the amount of YE primarily determines the maximum biomass that can be achieved. Additionally, an increment in the initial YE concentration did not alter the SA titer, and supports the observation that SA is mainly being produced from glucose. The direct relation between the initial YE concentration and the maximum biomass generated, regardless of the initial Glc concentration tested in these growth conditions, suggests that one or more limiting nutrients are being supplied by the YE. It would also appear that such nutrients cannot be synthesized from Glc, hence their depletion from YE limits growth long before Glc is exhausted. It is expected that the aromatic amino acids and vitamins present in the YE that are needed to counteract AR36 auxotrophy will become limiting; however, other compounds in this complex media may also play a role in growth limitation over time.

For a starting YE concentration of 30 g/L, a total of 106 g/L of Glc and 43 g/L of SA were consumed and produced, respectively, in approximately half the time than the fermentation with 15 g/L of YE. With 30 g/L of YE, the Qs_global_ and Qp_global_ increased twofold, in comparison with the fermentations with 15 g/L of YE, even though the SA titer remained unchanged (Table 3). Since the biomass also increased twofold, the calculated qp and qs were similar between the three experiments, both in exponential and stationary phases, exhibiting the metabolic robustness of the engineered strain under the tested conditions.

In addition, the results showed that an increase in YE concentration did not increase considerably the concentration of SA pathway intermediates (Figure 5c). In this respect, it has been acknowledged that the presence of high quantities of pathway intermediates can negatively impact the recovery of SA from the fermentation broth [7,38]. This concern has directed some efforts into the subject, leading to the testing of culture conditions, genetic backgrounds, and the use of non-metabolizable glucose analogs, as attempts to minimize byproduct generation [39].

In these experiments, a high proportion of SA relative to byproducts was detected without applying any further modification to the strain or process. The concentration of each pathway intermediate was compared against the sum of all aromatic intermediates, and their percentages were used to calculate the molar ratio of SA to each byproduct at the end of the fermentations (Figure 6). The ratio of SA turned out to be higher than 10 for DHS, QA, or DAHP, and higher than 40 for GA or DHQ for all the substrate concentrations tested. Remarkably, in all the conditions the obtained SA yields were close to 50% of the theoretical maximum and the yields of total aromatic compounds (TAC) were above 60% of the theoretical maximum, estimated as 0.86 mol_TAC_/mol_Glc_ (see Methods and Figure 6). This reflects the efficient redirection of glucose towards the AAA pathway in strain AR36, even when using high-glucose batch cultures. The ratio of SA to byproducts, as well as the obtained SA and TAC yields are fairly constant for all the conditions evaluated, and represent to our knowledge the highest reported values for a SA production fermentation process. These improvements can be justified by taking into account that the platform present in the engineered strain allows a more homogeneous expression of the necessary enzymes on an efficient genetic background. This, in contrast with other expression systems where the required genes are expressed from separate plasmids, under different promoters, or in strains not optimized for efficient use of high levels of Glc. In addition to the advantages concerning the dynamics of gene expression, the fact that IPTG is not needed to induce the Aro6 operon represents an important economical benefit for the production process, since the high price of IPTG restricts its use in large-scale fermentations.

### Insights on the glycolytic and acetate metabolisms of strain AR36 by RT-qPCR

To gain a deeper insight of the metabolic changes induced by the constitutive expression of the Aro6 synthetic operon in strain AR36, transcript levels of several genes were measured at three different growth stages in cultures with 50 g/L of Glc and 15 g/L of YE. As detailed in Methods, data obtained from early exponential phase (EE), late exponential phase (LE), and stationary phase (ST) were normalized against the values measured from strain AR3e at EE, grown under the same culture conditions.

**Figure 7:**
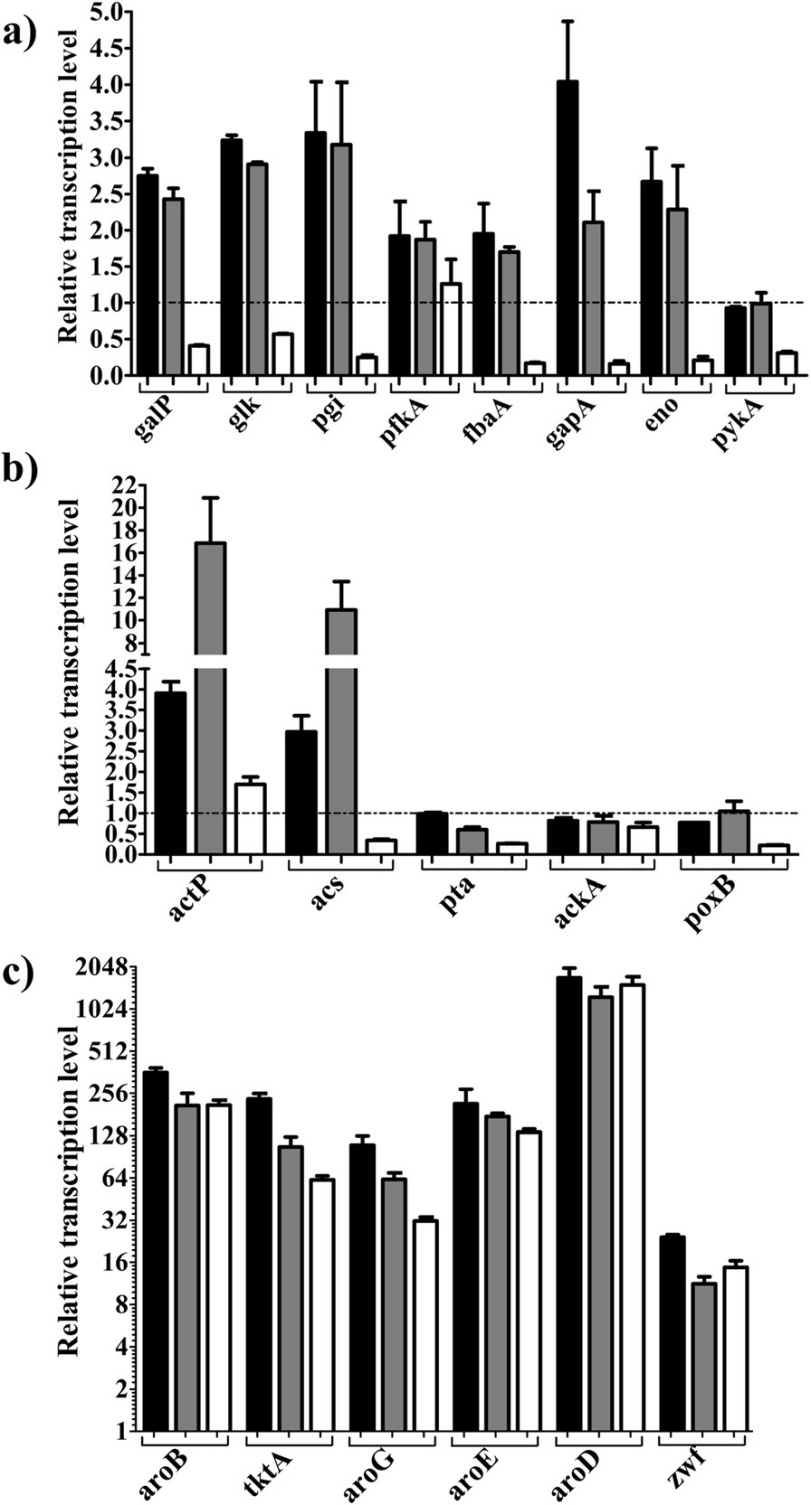
**Transcriptional changes resulting from the expression of Aro6 synthetic operon in strain AR36.** For comparison, the transcription level of each gene was determined at three different points in the growth curve of strain AR36, grown with 50 g/L of Glc and 15 g/L of YE (see Figure 4a). All data were normalized against the values obtained from strain AR3e at early exponential growth phase. **a)** Genes coding for glycolytic enzymes; **b)** genes involved in acetate assimilation and biosynthesis; **c)** genes comprised in the synthetic Aro6 operon (see Figure 1 and Figure 2). Black bars: early exponential phase; gray bars: late exponential phase; white bars: stationary phase. Error bars represent standard deviation.

The results indicate that the presence and expression of the operon in strain AR36 increases the transcriptional levels of several genes coding for glycolytic enzymes during the EE and LE phases (Figure 1 and Figure 7a). The rise in expression of genes *galP* and *glk* is particularly interesting because it has been reported that their products control the import and phosphorylation of glucose in PB12, the parental strain of AR36 [21,25]. Furthermore, there is a significant increase in the transcriptional levels of *pgi* and *eno*, but not *pykA.* These changes may translate into higher availability of PEP and fructose 6-P (which can be directly converted into E4P by plasmid-encoded transketolase), increasing the yield of aromatic compounds. We theorize that the observed upregulation of glycolytic genes in strain AR36 could be one of the consequences to low levels of some glycolytic intermediates (glucose 6-phosphate, fructose 6-phosphate and PEP), caused by the strong and constitutive expression of the operon-encoded enzymes that consume these metabolites.

On the other side, the transcriptional levels of genes coding for enzymes involved in acetate biosynthesis (*poxB*, *ackA* and *pta*) were not modified by the presence of the synthetic operon, while *actP* and *acs*, coding for enzymes involved in acetate assimilation, were strongly upregulated in the EE and LE phases (Figure 7b). Upregulation of *actP* and *acs* genes has also been detected in the exponential growth phase in the parental strain PB12 that is capable of co-utilizing Glc and acetate in minimal medium [21]. These findings correlate with the low levels of acetate in the assayed growth condition (Figure 4a). Importantly, the transcriptional values of these genes involved in acetate assimilation were low in ST phase (Figure 7b). If this response is representative of the other growth conditions used, it could partially explain the acetate accumulation observed in fermentations with 100 g/L of Glc, which consume higher amounts of Glc during stationary phase (Figure 4b and Figure 4c). These results highlight *actP* and *acs* as potential gene targets to artificially increase their expression in late culture stages, taking advantage of the expected capabilities of strain AR36 to utilize simultaneously Glc and acetate, present in its parental strain PB12 [21,40].

The genes present in the synthetic operon showed very strong expression levels (even in stationary phase), reflecting the constitutive nature of the promoter and high copy number of the plasmid (Figure 7c). These results correlate with the uninterrupted Glc consumption and SA production observed during the entire fermentation (Figure 4a), suggesting that the enzymes coded by the genes in the operon are present throughout the cultivation time. It can be seen in Figure 7c that the transcript levels of *aroD* and *zwf* are comparatively higher and lower, respectively, than the other four genes in the operon. This observation should be taken with caution because the six genes in the operon are being compared to the ones present in the chromosome of reference strain AR3e. Since the values obtained for the six genes are not normalized between them, variations amongst their chromosomal expression in strain AR3e can alter the relative comparisons with strain AR36. Nevertheless, the transcriptomic data is consistent with the high ratio of SA to aromatic intermediates obtained in the tested conditions, which is to be expected if all the genes in the operon were adequately expressed. Together with kinetic and stoichiometric data, these results highlight the benefits of employing a constitutively-expressed synthetic operon as an alternate strategy to increase the yield of SA from Glc in an evolved strain that lacks PTS and *pykF*.

## Conclusions

*E. coli* is the microorganism that has given the best results for SA production, with engineered strains that can accumulate up to 85 g/L using 10 L fermentors in fed-batch processes [10]. In this report, we showed that the constitutive and synchronous expression of a six-gene synthetic operon, in a laboratory-evolved strain bearing simultaneous PTS and *pykF* inactivations, resulted in a competitive process for the production of SA. The expression of Aro6 operon in the PTS^-^*pykF*^-^ derivative resulted in higher Qp_global_ and similar Qs_global_ than its PTS^-^*pykF*^+^ counterpart. In addition, the Glc consumption and SA production profiles of strain AR36 are consistent with the observed increase in transcription levels of glycolytic genes as a response to the constitutive expression of the operon in this strain. These features translated into significant improvements in growth and production parameters in strain AR36 (producing 0.74 gSA/L*h with 54% yield, using 50 g/L of Glc + 15 g/L of YE), compared to the PTS^-^*pykF*^-^ strain reported by Escalante et al. in 2010 (producing 0.11 gSA/L*h with 26% yield, using 25 g/L of Glc + 15 g/L YE).

Albeit fed-batch fermentations in the past have given the best results with respect to SA production, here we report that under the appropriate conditions, batch cultures of strain AR36 with an initially high concentration of Glc can also be efficiently used to produce SA. However, the production profiles obtained suggest that fed-batch fermentations could also yield good results with this strain. The fact that the SA production ceases only when the glucose is exhausted suggests that higher titers could be achieved by adequate Glc feeding strategies and improvements in the acetate uptake capabilities of this strain, considering that it lacks PTS and could co-utilize Glc and acetate in these growing conditions. The fermentations reported here yielded an elevated ratio of SA to other byproducts of the pathway (10 times more SA than the main byproducts generated). Besides increasing the SA yield, this behavior is relevant for purifying SA from the culture broth. In fact, preliminary experiments concerning SA purification from the broth obtained from these cultures, resulted in an almost quantitative purification process (unpublished results). Furthermore, the highest yield of total aromatic compounds obtained represents 67% of the theoretical maximum, demonstrating the efficient redirection of carbon to the AAA pathway by strain AR36. Nevertheless, the relatively low cellular concentration present in the cultures, even when administering high concentrations of YE, represents a significant problem to this production system because it restricts the productivity of the process. Other strategies need to be utilized in order to increase the biomass concentration without increasing the supplemented YE, which will constitute an important improvement for scaling-up the process. Minimizing the metabolic load imposed by a high-copy plasmid while maintaining a sufficient gene dosage of the operon, should improve the distribution of resources that are directed towards biomass generation and SA production.

## Methods

### Construction of *Escherichia coli* derivatives and plasmids

The laboratory-evolved strain PB12, a derivative of PB11 (obtained by the inactivation of PTS in strain JM101 [41]), was the receptor of the genetic modifications described in this work [21,22,24]. The strains and plasmids used in this report are listed in Table 1. The chromosomal inactivations of *aroK*, *aroL*, and *lacI* genes were performed sequentially by homologous recombination of PCR products [42]. In all cases, plasmid pKD3 was used as a PCR template in conjunction with tailored oligonucleotides containing 45 bp homology with the target chromosomal sequence (Additional file 2). Plasmid pKD46 expressed the Red recombinase system of bacteriophage lambda, and plasmid pCP20 allowed removal of the chloramphenicol resistance cassette after each event [43]. Every step was verified by PCR, identifying the clones that presented the expected amplicon sizes when using different sets of oligonucleotides and chromosomal DNA as a template (Additional file 2). The *pykF* inactivated gene was transduced to PB12 *aroK*^-^*aroL*^-^ using a P1 phage lysate obtained from strain PB28 (*pykF*::gen) [44]. Transductants were selected on gentamycin plates (10 μg/ml), and the inactivation was confirmed by PCR.

The construction of the Aro6 operon and expression vector was accomplished in several steps (Figure 2 and Additional file 3). First, *aroB, aroG*^fbr^, *tktA* and *aroE* genes were amplified by PCR using Pfu DNA polymerase and ligated sequentially into the polylinker of plasmid pBRINT-Ts Cm [45]. Chromosomal DNA from strain JM101 was used as a template for amplification of the required genes, with the exception of *aroG*^fbr^, which was amplified from plasmid pJLB*aroG*^fbr^*tktA*[46]. Different sets of oligonucleotides were employed for the amplification of each gene (Additional file 2), which also generated flanking restriction sites and consensus Shine-Dalgarno sequences (AGGAGG) situated 8 bp upstream of the start of each coding sequence. The PCR products were inserted into the polylinker in the following order: *aroB* in the *SmaI* site, *aroG*^fbr^ in the *XhoI* site, *tktA* in the *EcoRV* site, and *aroE* in the *ApaI* site. Simultaneously, plasmid pTrc327par*lacI*^+^ (Additional file 3) was built by ligating a PCR-amplified fragment containing the *lacI* gene, Trc promoter, polylinker, and transcriptional terminators of pTrc99A [31], into the *ScaI* and *PvuI* sites of pBR327par [32]. The 4-gene operon present in plasmid pBRINT-Ts Cm was amplified by PCR with a unique set of oligonucleotides (Additional file 1) and ligated into pTrc327par*lacI*^+^ after digesting both with *SacI* and *XbaI*. Later, *aroD* was amplified by PCR (flanked by *NheI* sites) and ligated into compatible *XbaI* site of pTrc327par*lacI*^+^. Because of our interest in expressing the operon in a constitutive manner, a *lacI*^-^ derivative of the initial pTrc327parlacI^+^ plasmid (without synthetic operon) was generated, and called pTrc327par (Additional file 3). The 5-gene operon was then transferred into *SacI* and *NcoI* sites of pTrc327par, giving rise to pTrcAro5. Finally, the *zwf* gene was inserted into the *XbaI* site, creating a 6-gene operon in pTrc327par. The resulting plasmid was named pTrcAro6 and transformed into AR2 and AR3 strains, generating AR26 and AR36, respectively (Table 1). The transformed strains were selected in LB plates supplemented with tetracycline (30 μg/ml).

Each step in the gene cloning and plasmid construction schemes was screened by endonuclease digestion and PCR, visualized with gel electrophoresis, and verified by DNA sequencing (3730, Perkin-Elmer/Applied Biosystems, USA). All the enzymes and reagents used in the molecular biology procedures were purchased from Fermentas (USA) and New England Biolabs (USA). When required, kits for the purification of PCR, plasmid, and agarose-embedded DNA were utilized (Roche, Switzerland). TOP10 cells (Invitrogen, USA) were used as a host for screening of DNA ligations during intermediate steps in vector construction.

### Cultivation media and growth conditions

#### Composition of production medium

SA production medium (adjusted to pH 7.0 with 10 N NaOH) contained per liter: K_2_HPO_4_ (7.5 g), KH_2_PO_4_ (7.5 g), citric acid monohydrate (2.1 g), ammonium iron (III) citrate (0.3 g), concentrated H_2_SO_4_ (1.2 ml), MgSO4 (0.64 g), CaCl_2_ (0.06 g), (NH_4_)_6_(Mo_7_O_24_) (0.0037 g), ZnSO_4_ (0.0029 g), H_3_BO_3_ (0.0247 g), CuSO_4_ (0.0025 g), MnCl_2_ (0.0158 g), CoCl_2_ (0.00129 g), thiamine (0.001 g), and betaine (0.234 g) as an osmoprotectant. Tetracycline (30 μg/ml) was added to inocula and cultures whenever needed for plasmid maintenance. Glucose (filter-sterilized) and yeast extract (added before autoclaving) were supplied at the concentrations indicated for each experiment. The glucose was purchased from JT Baker (USA) and the autolysed yeast extract from BD Difco (USA).

#### Shake flask cultures

The inoculum preparation for the shake flask cultures started by the addition of 1 ml frozen aliquots to 250 ml shake flasks containing 25 ml of production medium supplemented with glucose (25 g/L) and yeast extract (15 g/L). The inoculum was grown at 37°C and 300 rpm until mid-exponential phase, and approximately 5% of the final volume was transferred to the test shake flasks and incubated under the same controlled conditions with media containing 15 g/L of glucose and 5 g/L of yeast extract. Cell growth was measured by monitoring the optical density at 600 nm (OD_600_) in a DU700 spectrophotometer (Beckman, USA), and samples were taken periodically, centrifuged, and the supernatant was stored at -20°C for metabolite analysis. These experiments were performed at least in triplicate. All cultures started at approximately 0.3 OD_600_.

#### Fermentor cultures

Batch cultures were performed at least in duplicate using 1 L autoclavable glass bioreactors (Applikon, The Netherlands) with 500 ml of working volume. Bioreactors were connected to an Applikon ADI 1010 BioController and ADI 1025 controllers to monitor temperature, pH, impeller speed, and dissolved oxygen tension (DOT). The pH was kept at 7.0 by the addition of H_3_PO_3_ (3.3%) and NH_4_OH (10%). DOT in the culture medium was maintained by a continuous supply of filtered air (1 vvm), and by manually controlling the impeller speed (ranging from 500 to 1000 rpm) to ensure that DOT was kept above 20% at all times. The inoculum preparation for the fermentors started by the addition of 1 ml frozen aliquots to 500 ml shake flasks containing 50 ml of production medium supplemented with glucose (25 g/L) and yeast extract (15 g/L). The strains were grown at 37°C and 300 rpm until mid-exponential phase and approximately 5% of the final volume was transferred from each inoculum to previously prepared bioreactors containing the production medium. All fermentations were performed in presence of tetracycline (30 μg/ml). Cell growth was measured by monitoring optical density at 600 nm (OD_600_) in a spectrophotometer (DU700, Beckman, USA), and samples were taken periodically, centrifuged, and the supernatant was stored at -20°C for metabolite analysis. All the fermentations started at approximately 0.3 OD_600_.

### Metabolite quantification

The supernatant from each sample was properly diluted and filtered through 0.45 μM nylon membranes. Shikimic acid (SA), 3-dehydroshikimic acid (DHS), 3-dehydroquinic acid (DHQ), quinic acid (QA), gallic acid (GA), acetic acid, and glucose (Glc) concentrations were determined by HPLC using a Waters system (600E quaternary pump, 717 automatic injector, 2410 refraction index, and 996 photodiode array detectors; USA) equipped with an Aminex HPX-87H column (300 × 7.8 mm; 9 μm; Bio-Rad, USA). The mobile phase was 5 mM H_2_SO_4_, with a flow rate of 0.5 ml/min, maintained at 50°C. 3-deoxy-D-arabinoheptulosonate 7-phosphate (DAHP) concentrations were determined colorimetrically by the thiobarbituric acid assay [47]. This method does not distinguish between DAHP and its unphosphorylated form, DAH, therefore in this work DAHP levels correspond to the sum of both compounds.

### Data analysis and calculations

The measured concentrations of metabolites and biomass were normalized to the starting volume conditions to account for changes derived from pH control in fermentors. Data from independent experiments were averaged and presented in the corresponding graphs, where the error bars indicate the standard deviation for each point. Biomass concentration (X) was determined with a calibration curve between dry cellular weight and OD_600_, resulting in the equation X = 0.3587*OD_600_. Specific growth rate (μ) was determined by linearly fitting the biomass concentration to time during exponential phase with the following equation: lnX = lnXo + μ*t (where t is time, and X_0_ is the biomass concentration at initial time), displaying R^2^values >0.97. The yield of SA from Glc (Y_SA/Glc_) was calculated with the average molar concentrations of SA and Glc, produced and consumed, respectively, at the point of highest SA concentration. The yield of total aromatic compounds from glucose (Y_TAC/Glc_) was calculated with the combined molar yields of DAHP, DHQ, DHS, SA, QA, and GA at the point of highest SA concentration. The maximum theoretical yield of aromatic compounds was previously estimated as 0.86 mol_TAC_/mol_Glc_ for a PTS^-^ strain growing on glucose as only carbon source [10]. The global volumetric SA productivity (Qp_global_) and the global volumetric Glc consumption rate (Qs_global_) were calculated taking into account the time needed to reach the maximum SA concentration. Besides the previous calculations, linearizations were made to obtain apparent biomass on substrate (Y_X/S_) and product on biomass (Y_P/X_) yields. Although these apparent yields do not take into account the yeast extract consumption, correlation values for linearizations in all experiments were found to be >0.95, allowing comparisons between them. These yields were used to calculate the specific productivity and specific consumption rate on the exponential phase (qp_exp_ and qs_exp_, respectively) with the following equations: qp_exp_ = Y_P/X_*μ; qs_exp_ = μ/Y_X/S_.

The volumetric productivity and volumetric Glc consumption rate in stationary phase were determined by linearization of the first concentration data points at this stage versus time. The volumetric rates were utilized for calculation of specific production and consumption rates at stationary phase (qp_sta_ and qs_sta_, respectively) by dividing them by the average biomass concentration.

### RNA extraction, cDNA synthesis and RT-qPCR analysis

Samples from batch fermentations of strain AR36 with 50 g/L of Glc and 15 g/L of YE were collected for RNA extraction at early exponential phase, EE (2 h ~ 1 OD_600_), late exponential phase, LE (8 h ~ 12 OD_600_), and stationary phase, ST (24 h ~ 17 OD_600_), to determine gene expression levels. For comparison of the data, samples from early exponential phase (3.5 h ~ 1 OD_600_) of strain AR3e (bearing an empty pTrc327par plasmid carrying only the tetracycline-resistance gene, Figure 2) cultured under the same conditions were also collected and processed. RNA was extracted using hot phenol equilibrated with water, and cDNA synthesis was performed using RevertAid H First Strand cDNA Synthesis kit (Fermentas, USA) and a mixture of specific DNA primers, as reported previously [21,24]. qPCR experiments were performed with the ABI Prism 7300 Real Time PCR System (Applied Biosystems, USA) using Maxima SYBRGreen PCR Master Mix (Fermentas, USA) and reaction conditions previously described [21,24]. The quantification technique used to compare data was the 2^-∆∆CT^ method [48] and the results were normalized using the *ihfB* gene as an internal control. The same reproducible expression level for this gene was detected in all the strains and conditions analyzed [24]. All qPCR experiments complied with the MIQE guidelines for publication of quantitative real-time PCR experiments [49]. Using cells from two separate fermentations, RNA extraction and cDNA synthesis reactions were performed for each biological replicate at the indicated times and the gene expression values were measured by triplicate for each sample. Average values were graphed, with error bars representing standard deviation. Standard deviation was less than 30% in all cases.

## Competing interests

The authors have declared no competing interests.

## Authors’ contributions

AR, JLB and FB conceived the study and designed the experiments. AR and JAM carried out all fermentation experiments and data analysis. JAM performed the calculation of fermentation parameters. NF carried out RNA extraction and RT-qPCR experiments. GH was involved in metabolite quantification and analysis. AR performed the molecular biology and strain construction procedures and wrote the manuscript. JAM, OTR, GG and FB critically revised the results and the manuscript. All authors have read and approved the final manuscript.

## Supplementary Material

Additional file 1 **Nucleotide sequence of the synthetic operon constructed in this work and present in plasmid pTrcAro6.** The *aroG*^fbr^ gene included in this construction was a gift from DuPont™-Genencor®, therefore its coding sequence cannot be disclosed. Each nucleotide of *aroG*^fbr^ is indicated with an “n” except for the ones corresponding to its start and stop codons. Click here for file

Additional file 2 Oligonucleotides utilized in this work. Click here for file

Additional file 3 Detailed scheme of the construction of plasmid pTrcAro6. Click here for file
