# Supplementary material for: Constitutive expression of selected genes from the pentose phosphate and aromatic pathways increases the shikimic acid yield in high-glucose batch cultures of an Escherichia coli strain lacking PTS and pykF
Source: Microb Cell Fact. 2013 Sep 30;12:86. doi: 10.1186/1475-2859-12-86 (PMC3852013; doi:10.1186/1475-2859-12-86)
Supplement: Additional file 1 — Nucleotide sequence of the synthetic operon constructed in this work and present in plasmid pTrcAro6. The aroGfbr gene included in this construction was a gift from DuPont™-Genencor®, therefore its coding sequence cannot be disclosed. Each nucleotide of aroGfbr is indicated with an “n” except for the ones corresponding to its start and stop codons. [file 1475-2859-12-86-S1.pdf]

1	gccgacatca	taacggttct	ggcaaatatt	ctgaaatgag	ctgttgacaa	ttaatcatcc
61	ggctcgtata	atgtgtggaa	ttgtgagcgg	ataacaattt	cacacaggaa	acagaccatg
121	gaggaggtgc	tgctatggag	aggattgtcg	ttactctcgg	ggaacgtagt	tacccaatta
181	ccatcgcatc	tggtttgttt	aatgaaccag	cttcattctt	accgctgaaa	tcgggcgagc
241	aggtcatgtt	ggtcaccaac	gaaaccctgg	ctcctctgta	tctcgataag	gtccgcggcg
301	tacttgaaca	ggcgggtgtt	aacgtcgata	gcgttatcct	ccctgacggc	gagcagtata
361	aaagcctggc	tgtactcgat	accgtcttta	cggcgttgtt	acaaaaaccg	catggtcgcg
421	atactacgct	ggtggcgctt	ggcggcggcg	tagtgggcga	tctgaccggc	ttcgcggcgg
481	cgagttatca	gcgcggtgtc	cgtttcattc	aagtcccgac	gacgttactg	tcgcaggtcg
541	attcctccgt	tggcggcaaa	actgcggtca	accatcccct	cggtaaaaac	atgattggcg
601	cgttctacca	acctgcttca	gtggtggtgg	atctcgactg	tctgaaaacg	cttcccccgc
661	gtgagttagc	gtcggggctg	gcagaagtca	tcaaatacgg	cattattctt	gacggtgcgt
721	tttttaactg	gctggaagag	aatctggatg	cgttgttgcg	tctggacggt	ccggcaatgg
781	cgtactgtat	tcgccgttgt	tgtgaactga	aggcagaagt	tgtcgccgcc	gacgagcgcg
841	aaaccgggtt	acgtgcttta	ctgaatctgg	gacacacctt	tggtcatgcc	attgaagctg
901	aaatggggta	tggcaattgg	ttacatggtg	aagcggtcgc	tgcgggtatg	gtgatggcgg
961	cgcggacgtc	ggaacgtctc	gggcagttta	gttctgccga	aacgcagcgt	attataaccc
1021	tgctcaagcg	ggctgggtta	ccggtcaatg	ggccgcgcga	aatgtccgcg	caggcgtatt
1081	taccgcatat	gctgcgtgac	aagaaagtcc	ttgcgggaga	gatgcgctta	attcttccgt
1141	tggcaattgg	taagagtgaa	gttcgcagcg	gcgtttcgca	cgagcttgtt	cttaacgcca
1201	ttgccgattg	tcaatcagcg	taagtcaagt	cagcgtcagg	aggggctgca	ggaattcgat
1261	gctacgatct	agctaggagg	gagtcaaaat	gtcctcacgt	aaagagcttg	ccaatgctat
1321	tcgtgcgctg	agcatggacg	cagtacagaa	agccaaatcc	ggtcacccgg	gtgcccctat
1381	gggtatggct	gacattgccg	aagtcctgtg	gcgtgatttc	ctgaaacaca	acccgcagaa
1441	tccgtcctgg	gctgaccgtg	accgcttcgt	gctgtccaac	ggccacggct	ccatgctgat
1501	ctacagcctg	ctgcacctca	ccggttacaa	tctgccgatg	gaagaactga	aaaacttccg
1561	tcagctgcac	tctaaaactc	cgggtcaccc	ggaagtgggt	tacaccgctg	gtgtggaaac
1621	caccaccggt	ccgctgggtc	agggtattgc	caacgcagtc	ggtatggcga	ttgcagaaaa
1681	aacgctggcg	gcgcagttta	accgtccggg	ccacggcatt	gtcgaccact	acacctacgc
1741	cttcatgggc	gacggctgca	tgatggaagg	catctcccac	gaagtttgct	ctctggcggg
1801	tacgctgaag	ctgggtaaac	tgattgcatt	ctacgatgac	aacggtattt	ctatcgatgg
1861	tcacgttgaa	ggctggttca	ccgacgacac	cgcaatgcgt	ttcgaagctt	acggctggca
1921	cgttattcgc	gacatcgacg	gtcatgacgc	ggcatctatc	aaacgcgcag	tagaagaagc
1981	gcgcgcagtg	actgacaaac	cttccctgct	gatgtgcaaa	accatcatcg	gtttcggttc
2041	cccgaacaaa	gccggtaccc	acgactccca	cggtgcgccg	ctgggcgacg	ctgaaattgc
2101	cctgacccgc	gaacaactgg	gctggaaata	tgcgccgttc	gaaatcccgt	ctgaaatcta
2161	tgctcagtgg	gatgcgaaag	aagcaggcca	ggcgaaagaa	tccgcatgga	acgagaaatt
2221	cgctgcttac	gcgaaagctt	atccgcagga	agccgctgaa	tttacccgcc	gtatgaaagg
2281	cgaaatgccg	tctgacttcg	acgctaaagc	gaaagagttc	atcgctaaac	tgcaggctaa
2341	tccggcgaaa	atcgccagcc	gtaaagcgtc	tcagaatgct	atcgaagcgt	tcggtccgct
2401	gttgccggaa	ttcctcggcg	gttctgctga	cctggcgccg	tctaacctga	ccctgtggtc
2461	tggttctaaa	gcaatcaacg	aagatgctgc	gggtaactac	atccactacg	gtgttcgcga
2521	gttcggtatg	accgcgattg	ctaacggtat	ctccctgcac	ggtggcttcc	tgccgtacac
2581	ctccaccttc	ctgatgttcg	tggaatacgc	acgtaacgcc	gtacgtatgg	ctgcgctgat
2641	gaaacagcgt	caggtgacgg	tttacaccca	cgactccatc	ggtctgggcg	aagacggccc
2701	gactcaccag	ccggttgagc	aggtcgcttc	tctgcgcgta	accccgaaca	tgtctacatg
2761	gcgtccgtgt	gaccaggttg	aatccgcggt	cgcgtggaaa	tacggtgttg	agcgtcagga
2821	cggcccgacc	gcactgatcc	tctcccgtca	gaacctggcg	cagcaggaac	gaactgaaga
2881	gcaactggca	aacatcgcgc	gcggtggtta	tgtgctgaaa	gactgcgccg	gtcagccgga
2941	actgattttc	atcgctaccg	gttcagaagt	tgaactggct	gttgctgcct	acgaaaaact
3001	gactgccgaa	ggcgtgaaag	cgcgcgtggt	gtccatgccg	tctaccgacg	catttgacaa
3061	gcaggatgct	gcttaccgtg	aatccgtact	gccgaaagcg	gttactgcac	gcgttgctgt
3121	agaagcgggt	attgctgact	actggtacaa	gtatgttggc	ctgaacggtg	ctatcgtcgg
3181	tatgaccacc	ttcggtgaat	ctgctccggc	agagctgctg	tttgaagagt	tcggcttcac
3241	tgttgataac	gttgttgcga	aagcaaaaga	actgctgtaa	catactgagt	tcgtaggagg
3301	atcaagctta	tcgataccgt	cgacctcgac	aggagggaac	agacatgnnn	nnnnnnnnnn
3361	nnnnnnnnnn	nnnnnnnnnn	nnnnnnnnnn	nnnnnnnnnn	nnnnnnnnnn	nnnnnnnnnn
3421	nnnnnnnnnn	nnnnnnnnnn	nnnnnnnnnn	nnnnnnnnnn	nnnnnnnnnn	nnnnnnnnnn

3481 nnnnnnnnnn nnnnnnnnnn nnnnnnnnnn nnnnnnnnnn nnnnnnnnnn nnnnnnnnnn
3541 nnnnnnnnnn nnnnnnnnnn nnnnnnnnnn nnnnnnnnnn nnnnnnnnnn nnnnnnnnnn
3601 nnnnnnnnnn nnnnnnnnnn nnnnnnnnnn nnnnnnnnnn nnnnnnnnnn nnnnnnnnnn
3661 nnnnnnnnnn nnnnnnnnnn nnnnnnnnnn nnnnnnnnnn nnnnnnnnnn nnnnnnnnnn
3721 nnnnnnnnnn nnnnnnnnnn nnnnnnnnnn nnnnnnnnnn nnnnnnnnnn nnnnnnnnnn
3781 nnnnnnnnnn nnnnnnnnnn nnnnnnnnnn nnnnnnnnnn nnnnnnnnnn nnnnnnnnnn
3841 nnnnnnnnnn nnnnnnnnnn nnnnnnnnnn nnnnnnnnnn nnnnnnnnnn nnnnnnnnnn
3901 nnnnnnnnnn nnnnnnnnnn nnnnnnnnnn nnnnnnnnnn nnnnnnnnnn nnnnnnnnnn
3961 nnnnnnnnnn nnnnnnnnnn nnnnnnnnnn nnnnnnnnnn nnnnnnnnnn nnnnnnnnnn
4021 nnnnnnnnnn nnnnnnnnnn nnnnnnnnnn nnnnnnnnnn nnnnnnnnnn nnnnnnnnnn
4081 nnnnnnnnnn nnnnnnnnnn nnnnnnnnnn nnnnnnnnnn nnnnnnnnnn nnnnnnnnnn
4141 nnnnnnnnnn nnnnnnnnnn nnnnnnnnnn nnnnnnnnnn nnnnnnnnnn nnnnnnnnnn
4201 nnnnnnnnnn nnnnnnnnnn nnnnnnnnnn nnnnnnnnnn nnnnnnnnnn nnnnnnnnnn
4261 nnnnnnnnnn nnnnnnnnnn nnnnnnnnnn nnnnnnnnnn nnnnnnnnnn nnnnnnnnnn
4321 nnnnnnnnnn nnnnnnnnnn nnnnnnnnnn nnnnnnnnnn nnnnnnnnnn nnnnnnnnnn
4381 nnnnnnnnnn nnnntaagtc gaggggggc ccaggaggca gataatggaa acctatgctg
4441 tttttggtaa tccgatagcc cacagcaaat cgccattcat tcatcagcaa tttgctcagc
4501 aactgactat tgaacatccc tatgggcgcg tgttggcacc catcaatgat ttcatcaaca
4561 cactaaacgc tttctttagt gctggtggta aaggtgcgaa tgtgacggtg ccttttaaag
4621 aagaggcttt tgccagagcg gatgagctta ctgaacgggc agcgttggct ggtgctgtta
4681 ataccctcat gcggttagaa gatggacgcc tgctgggtga caataccgat ggtgtaggct
4741 tgttaagcga tctggaacgt ctgtctttta tccgccctgg tttacgtatt ctgcatatcg
4801 gcgctggtgg agcatctcgc ggcgtactac tgccactcct ttccctggac tgtgcggtga
4861 caataactaa tcggacggta tcccgcgcgg aagagttggc taaattgttt gcgcacactg
4921 gcagtattca ggcgttgagt atggacgaac tggaaggtca tgagtttgat ctcattatta
4981 atgcaacatc cagtggcatc agtggtgata ttccggcgat cccgtcatcg ctcattcatc
5041 caggcattta ttgctatgac atgttctatc agaaaggaaa aactcctttt ctggcatggt
5101 gtgagcagcg aggctcaaag cgtaatgctg atggtttagg aatgctggtg gcacaggcgg
5161 ctcatgcctt tcttctctgg cacggtgttc tgcctgacgt agaaccagtt ataaagcaat
5221 tgcaggagga attgtccgcg tgaatcagta tctagcagga ggcagataat gaaaaccgta
5281 actgtaaaag atctcgtcat tggtacgggc gcacctaaaa tcatcgtctc gctgatggcg
5341 aaagatatcg ccagcgtgaa atccgaagct ctcgcctatc gtgaagcgga ctttgatatt
5401 ctggaatggc gtgtggacca ctatgccgac ctctccaatg tggagtctgt catggcggca
5461 gcaaaaattc tccgtgagac catgccagaa aaaccgctgc tgtttacctt ccgcagtgcc
5521 aaagaaggcg gcgagcaggc gatttccacc gaggcttata ttgcactcaa tcgtgcagcc
5581 atcgacagcg gcctggttga tatgatcgat ctggagttat ttaccggtga tgatcaggtt
5641 aaagaaaccg tcgcctacgc ccacgcgcat gatgtgaaag tagtcatgtc caaccatgac
5701 ttccataaaa cgccggaagc cgaagaaatc attgcccgtc tgcgcaaaat gcaatccttc
5761 gacgccgata ttcctaagat tgcgctgatg ccgcaaagta ccagcgatgt gctgacgttg
5821 cttgccgcga ccctggagat gcaggagcag tatgccgatc gtccaattat cacgatgtcg
5881 atggcaaaaa ctggcgtaat ttctcgtctg gctggtgaag tatttggctc ggcggcaact
5941 tttggtgcgg taaaaaaagc gtctgcgcca gggcaaatct cggtaaatga tttgcgcacg
6001 gtattaacta ttttacacca ggcataagag ctcggtaccc ggggatcctc tagaaggagg
6061 ctcgcactat ggcggtaacg caaacagccc aggcctgtga cctggtcatt ttcggcgcga
6121 aaggcgacct tgcgcgtcgt aaattgctgc cttccctgta tcaactggaa aaagccggtc
6181 agctcaaccc ggacacccgg attatcggcg tagggcgtgc tgactgggat aaagcggcat
6241 ataccaaagt tgtccgcgag gcgctcgaaa ctttcatgaa agaaaccatt gatgaaggtt
6301 tatgggacac cctgagtgca cgtctggatt tttgtaatct cgatgtcaat gacactgctg
6361 cattcagccg tctcggcgcg atgctggatc aaaaaaatcg tatcaccatt aactactttg
6421 ccatgccgcc cagcactttt ggcgcaattt gcaaagggct tggcgaggca aaactgaatg
6481 ctaaaccggc acgcgtagtc atggagaaac cgctggggac gtcgctggcg acctcgcagg
6541 aaatcaatga tcaggttggc gaatacttcg aggagtgcca ggtttaccgt atcgaccact
6601 atcttggtaa agaaacggtg ctgaacctgt tggcgctgcg ttttgctaac tccctgtttg
6661 tgaataactg ggacaatcgc accattgatc atgttgagat taccgtggca gaagaagtgg
6721 ggatcgaagg gcgctggggc tattttgata aagccggtca gatgcgcgac atgatccaga
6781 accacctgct gcaaattctt tgcatgattg cgatgtctcc gccgtctgac ctgagcgcag
6841 acagcatccg cgatgaaaaa gtgaaagtac tgaagtctct gcgccgcatc gaccgctcca
6901 acgtacgcga aaaaaccgta cgcgggcaat atactgcggg cttcgcccag ggcaaaaaag

6961 tgccgggata tctggaagaa gagggcgcga acaagagcag caatacagaa actttcgtgg
7021 cgatccgcgt cgacattgat aactggcgct gggccggtgt gccattctac ctgcgtactg
7081 gtaaacgtct gccgaccaaa tgttctgaag tcgtggtcta tttcaaaaca cctgaactga
7141 atctgtttaa agaatcgtgg caggatctgc cgcagaataa actgactatc cgtctgcaac
7201 ctgatgaagg cgtggatatc caggtactga ataaagttcc tggccttgac cacaaacata
7261 acctgcaaat caccaagctg gatctgagct attcagaaac ctttaatcag acgcatctgg
7321 cggatgccta tgaacgtttg ctgctggaaa ccatgcgtgg tattcaggca ctgtttgtac
7381 gtcgcgacga agtggaagaa gcctggaaat gggtagactc cattactgag gcgtgggcga
7441 tggacaatga tgcgccgaaa ccgtatcagg ccggaacctg ggacccgtt gcctcggtgg
7501 cgatgattac ccgtgatggt cgttcctgga atgagtttga gtaatctaga gtcgacctgc
7561 aggcatgcaa gcttggctgt tttggcggat gagagaagat tttcagcctg atacagatta
7621 aatcagaacg cagaagcggt ctgataaaac agaatttgcc tggcggcagt agcgcggtgg
7681 tcccacctga ccccatgccg aactcagaag tgaaacgccg tagcgccgat ggtagtgtgg
7741 ggtctcccca tgcgagagta gggaactgcc aggcatcaaa taaaacgaaa ggctcagtcg
7801 aaagactggg cctttcgttt tatctgttgt ttgtcggtga acgctctcct gagtaggaca
7861 aatccgccgg gagcggattt gaacgttgcg aagcaacggc ccggagggtg gcgggcagga
7921 cgcccgccat aaactgccag gcatcaaatt aagcagaagg ccatcctgac ggatggactc
7981 aaccaagtca ttctgagaat agtgtatgcg gcgaccgagt tg
