# Supplementary material for: Constitutive expression of selected genes from the pentose phosphate and aromatic pathways increases the shikimic acid yield in high-glucose batch cultures of an Escherichia coli strain lacking PTS and pykF
Source: Microb Cell Fact. 2013 Sep 30;12:86. doi: 10.1186/1475-2859-12-86 (PMC3852013; doi:10.1186/1475-2859-12-86)
Supplement: Additional file 2 — Oligonucleotides utilized in this work. [file 1475-2859-12-86-S2.pdf]

Name (size in bp)	Sequence (5'- 3')	Function
aroKcatFw (67)	AATAGTCTTAGTAGTACCGAAAAAATGGCAGAGAAACGCAATA TCTGTGTAGGCTGGAGCTGCTTCG	Amplification of cat gene from pDK3 with homologous region for aroK
aroKcatRv (65)	AGACGAGTGTATATAAAGCCAGAATTAGTTGCTTTCCAGCATGT GATGGGAATTAGCCATGGTCC	Amplification of cat gene from pDK3 with homologous region for aroK
aroLcatFw (66)	GCGACCTATTGGGGAAAACCCACGATGACACAACCTCTTTTTCT GTGTGTAGGCTGGAGCTGCTTC	Amplification of cat gene from pDK3 with homologous region for aroL
aroLcatRv (65)	ACGTTAAGTATAGGCGCTCGAAAATCAACAATTGATCGTCTGTG CATGGGAATTAGCCATGGTCC	Amplification of cat gene from pDK3 with homologous region for aroL
lacIcatFw (65)	TGTCTTCGGTATCGTCGTATCCCACTACCGAGATATCCGCACCAA GTGTAGGCTGGAGCTGCTTC	Amplification of cat gene from pDK3 with homologous region for lacI
lacIcatRv (65)	GCTGATTGGCGTTGCCACCTCCAGTCTGGCCCTGCACGCGCCGTC ATGGGAATTAGCCATGGTCC	Amplification of cat gene from pDK3 with homologous region for lacI
-500aroK5' (20)	CATTCCCTGGTTCGGGCAAT	Verification of aroK inactivation
+500aroK3' (20)	GGGATGGTTGACCGCAGTTT	Verification of aroK inactivation
-500aroL5' (20)	TTGTCCGGCAGTGTGAAGCG	Verification of aroL inactivation
+500aroL3' (20)	TTACCGGGCAAGTGTGAAGC	Verification of aroL inactivation
lacI5'verif (21)	GCAACAGCTGATTGCCCTTCA	Verification of lacI inactivation
lacI3'verif (23)	AAGCGGCGATGGCGGAGCTGAAT	Verification of lacI inactivation
-700pykF5' (20)	CAGCTCACTGCGCTGAATGC	Verification of pykF inactivation
+800pykF3' (20)	CACTATCGGCAGAAGAACAG	Verification of pykF inactivation
aroB5' (41)	CATACTGAGTTCGTAGGAGGGTCGCGTTATGGAGAGGATTG	Amplification of aroB for cloning
aroB3' (40)	CCTCCTGACGCTGACTTGACTTACGCTGATTGACAATCGG	Amplification of aroB for cloning
aroG5' (36)	GCGCGCGTCGACAGGAGGGAACAGACATGAATTATC	Amplification of $aroG^{fbr}$ for cloning
aroG3' (32)	GGTACGGTCGACTTACCCGCGACGCGCTTTTA	Amplification of $aroG^{fbr}$ for cloning
tktA5' (39)	GCTACGATCTAGCTAGGAGGGAGTCAAAATGTCCTCACG	Amplification of tktA for cloning
tktA3' (40)	CCTCCTACGAACTCAGTATGTTACAGCAGTTCTTTTGCTT	Amplification of tktA for cloning
aroE5' (47)	GCGTCGAGGGCCCAGGAGGCAGATAATGGAAACCTATGCTGTTT TTG	Amplification of aroE for cloning
aroE3' (40)	ATCTAGCGGGCCCTACTGATTCACGCGGACAATTCCTCCT	Amplification of aroE for cloning
aroD5' (47)	GCGCCGCGCTAGCAGGAGGCAGATAATGAAAACCGTAACTGTA AAAG	Amplification of aroD for cloning
aroD3' (41)	CGCTGGTCTCGCGGCTAGCTTATGCCTGGTGTAAAATAGTT	Amplification of aroD for cloning
zwf5' (47)	GCTATGCTCTAGAAGGAGGCTCGCACTATGGCGGTAACGCAAAC AGC	Amplification of zwf for cloning
zwf3' (38)	CGCGACGCTCTAGATTACTCAAACTCATTCCAGGAACG	Amplification of zwf for cloning
5'trc (46)	GTCATCACCGATCGATATCTGCAGGTGGTGAACCAGGCCAGCCA CG	Cloning of fragment from pTrc99A into pBR327par
3'trc (27)	CCATCCGTCAGGATGGCCTTCTGCTTA	Cloning of fragment from pTrc99A into pBR327par
5'Aro4 (34)	CATGCAGAGCTCATGGAGAGGATTGTCGTTACTC	Cloning of 4-gene operon into pTrc327parlacI+
3'Aro4 (34)	GCCGTGTCTAGATACTGATTCACGCGGACAATTC	Cloning of 4-gene operon into pTrc327parlacI+
SHK5'A (50)	GTAGTACGCCATGGAGGAGGTCGCTGCTATGGAGAGGATTGTCG TTACTC	Transfer of 5-gene operon to pTrc327par
SHK3'A (36)	CGCTGCGCGAGCTCTTATGCCTGGTGTAAAATAGTT	Transfer of 5-gene operon to pTrc327par
SHK5'B (22)	GCCGACATCATAACGGTTCTGG	Amplification of entire Aro6 operon with promoter and terminators
SHK3'B (23)	CAACTCGGTCGCCGCATACACTA	Amplification of entire Aro6 operon with promoter and terminators
