# Supplementary figures and images for: Constitutive expression of selected genes from the pentose phosphate and aromatic pathways increases the shikimic acid yield in high-glucose batch cultures of an Escherichia coli strain lacking PTS and pykF
Source: Microb Cell Fact. 2013 Sep 30;12:86. doi: 10.1186/1475-2859-12-86 (PMC3852013; doi:10.1186/1475-2859-12-86)

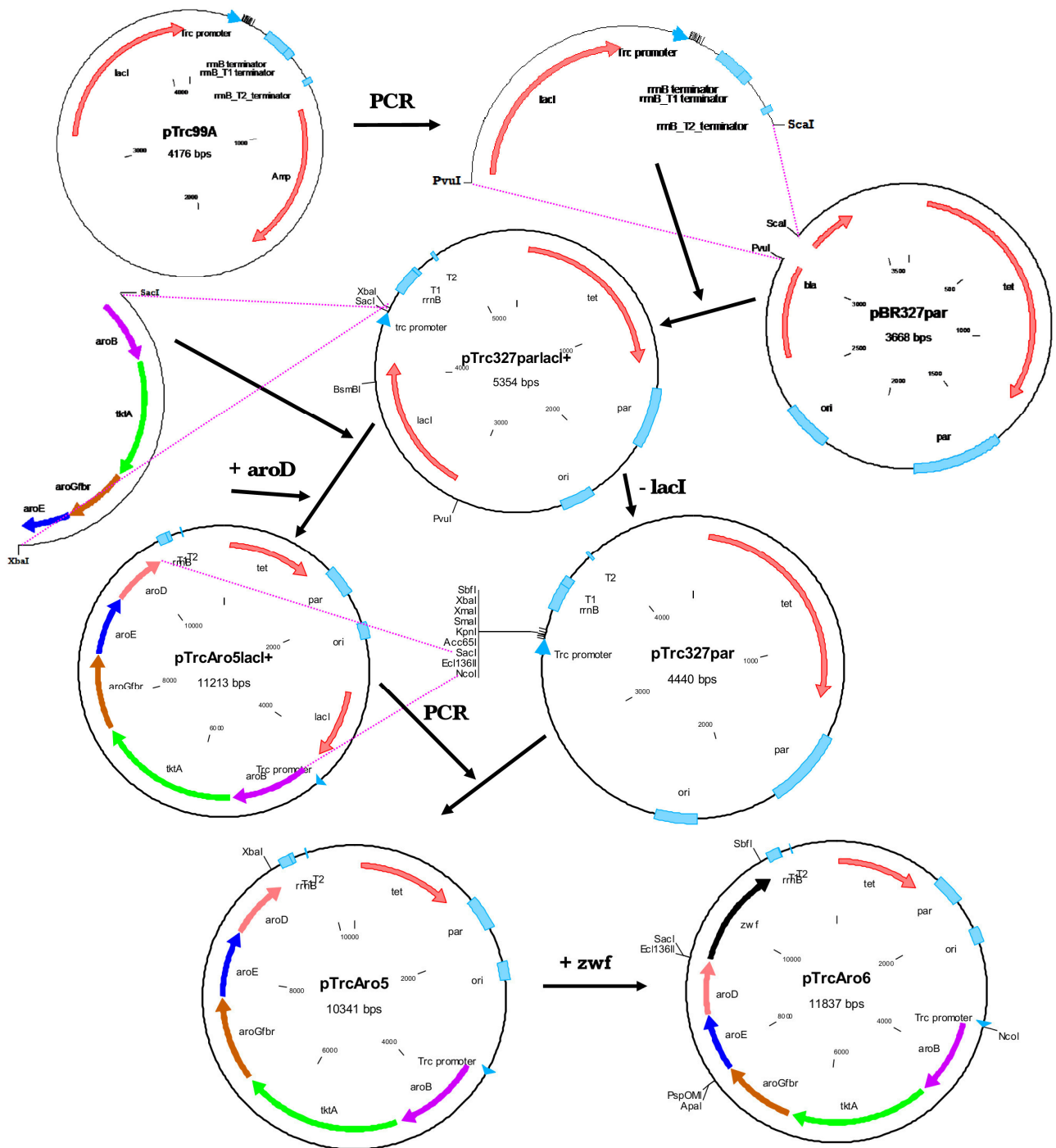

Supplement: Additional file 3 — Detailed scheme of the construction of plasmid pTrcAro6. [file 1475-2859-12-86-S3.pdf]
